# Identification of beneficial populations for targeted-immunotherapy combinations: tailoring later-line care for patients with pMMR/MSS metastatic colorectal cancer

**DOI:** 10.3389/fimmu.2024.1462346

**Published:** 2024-10-24

**Authors:** Dan Li, Hui Jin, Yan Liu, Jiayin Liu, Xue Zhang, Long Wang, Zhisong Fan, Li Feng, Jing Zuo, Jing Han, Yudong Wang

**Affiliations:** Department of Medical Oncology, The Fourth Hospital of Hebei Medical University, Shijiazhuang, China

**Keywords:** microsatellite stable metastatic colorectal cancer, third-line or beyond, real-world, targeted-immunotherapy combination, beneficial population

## Abstract

**Objective:**

This study explores the benefits of targeted-immunotherapy combination in third-line or beyond treatment for microsatellite stable (MSS) metastatic colorectal cancer (mCRC) in a real-world setting.

**Methods:**

Patients with MSS mCRC who were treated with either a targeted-immunotherapy combination or targeted therapy alone in the third-line or beyond setting at our hospital from August 2018 to August 2022 were included in the study. Inclusion criteria comprised patients treated with targeted therapy alone or in combination with immunotherapy. Effectiveness was compared between treatments, and patients with the potential to benefit from targeted-immunotherapy combination were identified.

**Results:**

Among 71 patients, 31 received targeted therapies alone (TT group) and 40 received a combination of targeted therapy and immunotherapy (TI group). The TI group had higher objective response rates (20% vs 3.2%) and disease control rates (82.5% vs 58.1%). The median progression-free survival was significantly better in the TI group (4.6 vs 4.1 months, P = 0.027). Liver metastasis was associated with poor prognosis, while patients with only lung metastases had the longest median progression-free survival of 12.3 months with combination therapy.

**Conclusion:**

The study indicates that targeted-immunotherapy combination offers more benefits than targeted therapy alone for MSS mCRC in the third-line or beyond setting.

## 1 Introduction

Colorectal cancer (CRC) is the third most commonly diagnosed cancer and the second leading cause of cancer-related deaths worldwide (1). The incidence of CRC in China, although historically significantly lower than in Western countries, has increased rapidly in recent years, making it the most common malignant tumor of the digestive system. According to the latest 2022 data on the cancer burden in China, CRC ranks second in incidence and fourth in mortality in the country (2). For metastatic CRC (mCRC), treatment options are limited after progression following standard front-line treatments, resulting in limited survival benefits (3–6). Further, in contrast to front-line therapy, the main goals of third-line or beyond treatment for this population are to control tumor progression, prolong survival, and improve quality of life (7).

In recent years, immunotherapy has emerged as a promising new approach for treating mCRC, especially for tumors with high microsatellite instability (MSI-H), including as a third-line treatment for MSI-H mCRC (8–10). However, MSI-H tumors account for only about 5% of cases (11), while the remaining 95% are microsatellite stable (MSS) CRCs. MSI-H tumors are characterized by a high mutational burden, which increases the presentation of neoantigens and enhances the infiltration of immune cells, leading to an “inflamed” microenvironment. This feature makes MSI-H tumors more responsive to immune checkpoint inhibitors. In contrast, MSS tumors typically exhibit a “cold” immune microenvironment, with a low mutational burden and minimal immunity, rendering them representative “cold tumors” (12). Immunotherapy appears to be ineffective against MSS tumors, with many exploratory studies having failed (13–15).

Given the synergistic effects of immunotherapy with anti-angiogenic therapy, several studies have evaluated the addition of programmed cell death protein 1 (PD-1) inhibitors to the standard anti-angiogenic monotherapy in patients with MSS mCRC. The phase Ib REGONIVO study evaluating nivolumab combined with regorafenib as third-line or beyond treatment enrolled 25 patients in the CRC cohort, with 24 patients having MSS tumors, and showed the encouraging anti-tumor activity. Among the 25 patients, the objective response rate (ORR) was 36% (with an ORR of 33% in MSS patients), the median progression-free survival (PFS) was 7.9 months, and the median overall survival (OS) was not reached (16). On the contrary, the phase II REGOMUNE trial combining avelumab with regorafenib, patients achieved only stable disease as the best response (17). The inconsistent data indicated that only a small fraction of patients might benefit from targeted-immunotherapy combination. It is important to note that studies exploring combination therapies were all single-arm designs, and little is known about comparisons of targeted-immunotherapy combination with standard targeted monotherapy in this patient population. Also, the effectiveness of this combination therapy in routine clinical practice remains uncertain. Here, we designed this retrospective study to compare the effectiveness of targeted-immunotherapy combination with targeted therapy alone in the third-line or beyond setting for MSS mCRC patients and to identify the potential beneficial population of combined targeted-immunotherapy.

## 2 Materials and methods

### 2.1 Patient population

Data on MSS mCRC patients who received third-line or beyond treatment at the Fourth Hospital of Hebei Medical University between August 2018 to August 2023 were retrospectively collected by reviewing electronic medical records. Patients with MSS mCRC who were treated with targeted therapy alone or in combination with immunotherapy as third-line or beyond therapy were included. Immunohistochemistry (IHC) staining of four kinds of MMR protein (MLH1, MSH2, MSH6, PMS2) or polymerase chain reaction (PCR) analysis of five microsatellite markers (BAT25, BAT26, D5S346, D2S123, D17S250) were used to determine MSS status of colorectal cancer patients. Patients diagnosed with MSI-H/dMMR status were excluded from the study. The demographic data, clinicopathological information, treatment records, imaging examination results, and survival outcomes were collected in detail from electronic medical records. This study was approved by the Ethics Committee of the Fourth Hospital of Hebei Medical University (approval number: 20230926) and was performed in accordance with the Declaration of Helsinki. This article is a retrospective study and has obtained ethical exemption.

### 2.2 Clinical data

The start date of third-line treatment was defined as the start date. The follow-up period was defined as the time from the date of initiation of third-line or beyond treatment until the data cut-off date (February 29, 2024), the last outpatient visit, or death. Baseline clinical characteristics were assessed either before or at the start of third-line or beyond treatment. After treatment, all patients underwent imaging examinations every two cycles (6 weeks) to evaluate clinical efficacy as per the Response Evaluation Criteria in Solid Tumors (RECIST) version 1.1. The ORR was defined as the proportion of patients whose best response was either complete response (CR) or partial response (PR). Disease control rate (DCR) was defined as the proportion of patients who achieved CR, PR, or stable disease (SD). PFS was defined as the time from the start of third-line or beyond treatment to the first recorded disease progression or death, whichever occurred first. OS was defined as the time from the start of third-line or beyond treatment to death from any cause.

### 2.3 Statistical analysis

All statistical analyses in this study were conducted using IBM SPSS Statistics version 27.0 (New York, USA). Categorical variables were summarized as number (percentage) and compared using the chi-squared test or Fisher’s exact test. Continuous variables were described with median and range. The OS and PFS were analyzed using the Kaplan-Meier method, and comparisons were made using the log-rank test. Additionally, univariate and multivariate Cox proportional hazards regression models were used to analyze potential risk characteristics. Hazard ratios (HRs) and the 95% confidence intervals (CIs) were estimated to quantify the strength of these associations. A p-value of <0.05 was considered statistically significant, and all tests were two-tailed.

## 3 Results

### 3.1 Baseline patient and clinical characteristics

Among the 71 patients included, 31 (43.7%) patients received targeted therapy (TT group) while 40 (56.3%) patients were treated with a combination of targeted therapy and immunotherapy (TI group) (**Table 1**). Both groups had a similar median age of 57 years, and the overall gender distribution showed more males (57.7%) than females (42.3%), with a notably higher percentage of males in the TT group (71.0%) than in the TI group (47.5%). Overall, most patients (87.3%) had an ECOG performance status of 0 or 1. The primary tumor site was distributed predominantly in the rectum (50.7%), followed by the left hemi-colon (35.2%) and the right hemi-colon (14.1%). Multiple metastatic sites were common (74.6%), with lung (64.8%), liver (53.5%), and lymph nodes (47.9%) being predominant. Regarding genetic mutations, KRAS or NRAS mutations were found in 40.8% of patients, and BRAF V600E mutations in 7%. There were no statistically significant differences between the TT and TI groups regarding baseline characteristics other than age.

**Table 1:** Baseline characteristics of patients.

Characteristics	Total (*n* = 71)	TT group (*n* = 31)	TI group (*n* = 40)	*P* value
**Age, years**				0.912
< 60, *n* (%)	43 (60.6)	19 (61.3)	24 (60.0)	
≥ 60, *n* (%)	28 (39.4)	12 (38.7)	16 (40.0)	
Median	57	57	57.5	
Range	29-77	29-72	34-77	
**Gender, *n* (%)**				0.047
Male	41 (57.7)	22 (71.0)	19 (47.5)	
Female	30 (42.3)	9 (29.0)	21 (52.5)	
**ECOG PS, *n* (%)**				
0-1	62 (87.3)	26 (83.9)	36 (90.0)	0.127
2	9 (12.7)	5 (16.1)	4 (10.0)	
**Primary tumor site, *n* (%)**				0.265
Right colon	10 (14.1)	6 (19.4)	4 (10.0)	
center colon	25 (35.2)	8 (25.8)	17 (42.5)	
Rectum	36 (50.7)	17 (54.8)	19 (47.5)	
**Stage at initial diagnosis, *n* (%)**				0.686
Initial diagnosis of stage IV	34 (47.9)	14 (45.2)	20 (50.0)	
Postoperative recurrence	37 (52.1)	17 (54.8)	20 (50.0)	
**Number of metastatic sites, *n* (%)**				0.530
Single	18 (25.4)	9 (29.0)	9 (22.5)	
Multiple (≥ 2)	53 (74.6)	22 (71.0)	31 (77.5)	
**Site of metastases, *n* (%)**				
Lymph node	34 (47.9)	15 (48.4)	19 (47.5)	0.941
Liver	38 (53.5)	18 (58.1)	20 (50.0)	0.499
Lung	46 (64.8)	22 (71.0)	24 (60.0)	0.337
Bone	6 (8.5)	2 (6.5)	4 (10.0)	0.918
Peritoneum	17 (23.9)	5 (16.1)	12 (30.0)	0.174
**RAS mutation status, *n* (%)**				0.909
KRAS, NRAS all wild type	30 (42.3)	14 (45.2)	16	
KRAS or NRAS mutant	29 (40.8)	12 (38.7)	17 (42.5)	
Unknown	12 (16.9)	5 (16.1)	7	
**BRAF mutation status, *n* (%)**				0.493
BRAF^V600E^ wild type	45 (63.4)	21 (67.7)	24 (60.0)	
BRAF^V600E^ mutant	5 (7.0)	1 (3.2)	4 (10.0)	
Unknown	21 (29.6)	9 (29.3)	12 (30.0)	

ECOG PS, Eastern Cooperative Oncology Group performance status; pMMR, mismatch repair proficient; dMMR, mismatch repair deficiency; MSI-H, high microsatellite instability; MSS, microsatellite stable; TT group, targeted therapy group; TI group, targeted-immunotherapy combination group.

As shown in **Table 2**, 25 (86.4%) and 31 (77.5%) patients in the TT and TI groups, respectively, received third-line treatment. In the third-line or beyond setting, regorafenib was the most commonly used targeted agent (64.5% in the TT group and 75.0% in the TT group), while in terms of immunotherapy in the TI group, camrelizumab was the dominant agent (65.0%).

**Table 2:** Prior systemic treatment regimens.

Treatment regimens	TT group (*n* = 31)	TI group (*n* = 40)	*P*
**First line, *n* (%)**			0.697
Chemotherapy	8(25.8)	12(30.0)	
Chemotherapy-targeted combination	23(74.2)	28(70.0)	
**Second line, *n* (%)**			0.146
Chemotherapy	7(22.6)	4(10.0)	
Chemotherapy-targeted combination	24(77.4)	36(90.0)	
**Third-line or beyond, *n* (%)**			0.747
Third-line therapy	25(80.6)	31(77.5)	
Beyond third-line therapy	6(19.4)	9(22.5)	
**Targeted drugs for third-line or beyond, *n* (%)**			0.337
Regorafenib	20(64.5)	30(75.0)	
Fruquintinib	11(35.5)	10(25.0)	
**Immune checkpoint inhibitors for third-line or beyond, *n* (%)**			–
Camrelizumab	–	26(65.0)	
Tislelizumab	–	5(12.5)	
Sintilimab	–	4(10.0)	
Others	–	5(12.5)	

TT group, targeted therapy group; TI group, targeted-immunotherapy combination group.

### 3.2 Efficacy

**Figure 1:**
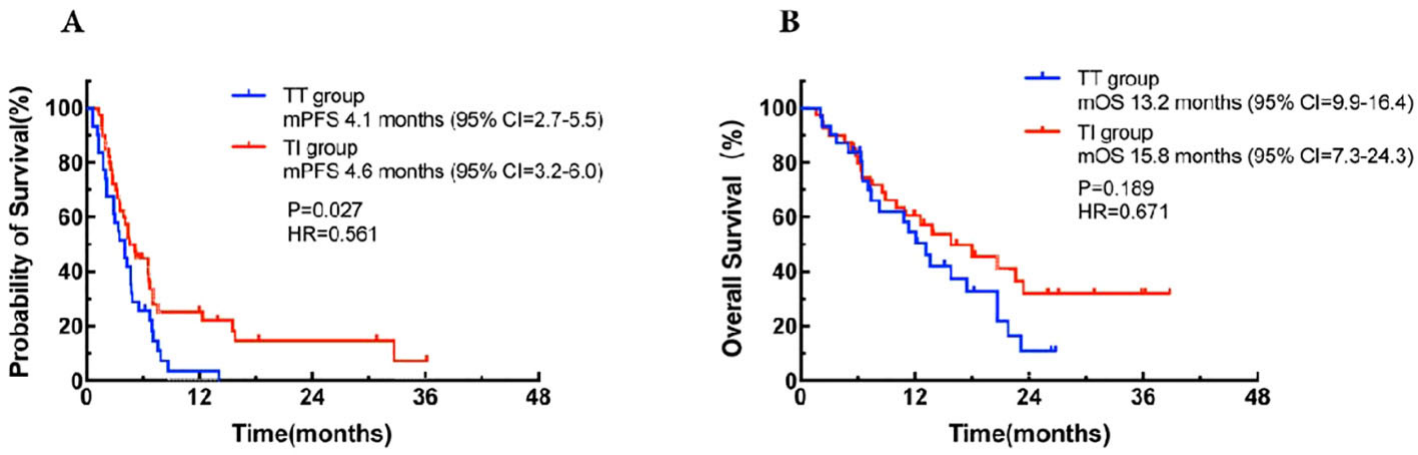
Kaplan-Meier curves for progression-free survival **(A)** and overall survival **(B)** in the TT group and the TI group and the TI group. CI, confidence interval; HR, hazard ration; mOS, median overall survival; mPFS, median progression-free survival; TT group, targeted therapy group; TI group, targeted-immunotherapy combination group.

A total of 71 patients were assessable for response. As shown in **Table 3**, there was a noticeable difference in the response to third-line or beyond treatment between the TT group and the TI group in patients with MSS mCRC. The ORR and DCR in the TI group were significantly higher than those in the TT group, with 20.0% vs 3.2% (odds ratio [OR] = 0.080, 95% CI: 0.023-0.275, *P* = 0.000) and 82.5% vs 58.1% (OR = 0.024, 95% CI: 0.008-0.074, *P* = 0.000), respectively. These findings suggest that the addition of immunotherapy to targeted therapy may improve the control of the disease in this patient population. For all the 71 patients regardless of treatment, the overall median PFS was 4.4 months (95% CI: 1.3-36.2) and the median OS was 13.8 months (95% CI: 1.6-38.8). Further, the median PFS was 4.1 months (95% CI: 2.7-5.5) in the TT group, while in the TI group, the corresponding value was 4.6 months (95% CI: 3.2-6.0), with a statistically significant difference between the two groups (HR = 0.561, 95% CI: 0.34-0.94, *P* = 0.027; **Figure 1A**). This demonstrates that in third-line or beyond setting, the combination of targeted therapy and immunotherapy may provide a longer PFS compared to monotherapy with targeted agents. In terms of OS, an improved trend was observed in the TI group as compared to that in the TT group (15.8 months [95% CI: 7.3-24.3] vs 13.2 months [95% CI: 9.9-16.4]), although with no statistically significant difference between the two groups (HR = 0.671, 95% CI: 0.37-1.21, *P* = 0.189; **Figure 1B**).

**Table 3:** Tumor response.

Tumor response, *n* (%)	Total (*n* = 71)	TT group (*n* = 31)	TI group (*n* = 40)	OR	95% CI		*P* value
					Lower	Upper	
CR	0 (0)	0 (0)	0 (0)				
PR	9 (12.7)	1 (3.2)	8 (20.0)				
SD	40 (56.3)	17 (54.8)	23 (57.5)				
PD	22 (31.0)	13 (41.9)	9 (22.5)				
ORR	9 (12.7)	1 (3.2)	8 (20.0)	0.080	0.023	0.275	0.000
DCR	51 (71.8)	18 (58.1)	33 (82.5)	0.024	0.008	0.074	0.000

CI, confidence interval; CR, complete response; DCR, disease control rate; EGFR, epidermal growth factor receptor; OR, odds ratio; ORR, overall response rate; PD, disease progression; PR, partial response; SD, stable disease; TT group, targeted therapy group; TI group, targeted-immunotherapy combination group.

Cox proportional hazards univariate analysis showed that apart from the number of metastatic sites, lymph node metastasis and liver metastasis, none of the other factors showed a significant association with PFS (**Table 4**); similarly, lymph node involvement and liver metastasis were also significantly associated with OS (**Table 5**). Multivariate analysis further identified liver metastasis as an independent prognostic factor for both PFS (HR = 0.407, 95% CI: 0.217-0.761, *P* = 0.005) and OS (HR = 0.386, 95% CI: 0.179-0.832, *P* = 0.015).

**Table 4:** Univariate and multivariate analysis of factors predicting PFS.

Variable	Univariate analysis				Multivariate analysis			
	HR	95% CI		*P*	HR	95% CI		*P*
		Lower	Upper			Lower	Upper	
Age, years								
≥ 60 vs < 60	0.865	0.519	1.441	0.577				
Gender								
Female vs male	0.614	0.355	1.060	0.080	1.333	0.763	2.327	0.313
Primary tumor site								
center colon vs right colon	0.854	0.378	1.927	0.704				
Rectum vs right colon	0.888	0.407	1.936	0.765				
Stage at initial diagnosis								
Postoperative recurrence vs initial diagnosis of stage IV	0.803	0.486	1.326	0.391				
Number of metastatic sites								
Single vs multiple (≥ 2)	0.546	0.299	0.994	0.048*	0.886	0.403	1.948	0.764
Lymph node metastasis								
No metastasis vs metastasis	0.569	0.340	0.950	0.031*	0.638	0.356	1.142	0.130
Liver metastasis								
No metastasis vs metastasis	0.378	0.231	0.649	0.000*	0.407	0.217	0.761	0.005*
Lung metastasis								
Metastasis vs no metastasis	0.739	0.442	1.235	0.249				
Bone metastasis								
Metastasis vs no metastasis	0.964	0.409	2.272	0.934				
Peritoneum metastasis								
No metastasis vs metastasis	0.941	0.531	1.668	0.836				
RAS status								
Mutant vs wild	0.827	0.478	1.431	0.497				
Unknown vs wild	0.967	0.477	1.961	0.926				
BRAF status								
Mutant vs wild	0.651	0.230	1.843	0.419				
Unknown vs wild	0.757	0.433	1.325	0.330				

CI, confidence interval; HR, hazard ratio; PFS, progression-free survival; TT group, targeted therapy group; TI group, targeted-immunotherapy combination group. * indicates statistical significance.

**Table 5:** Univariate and multivariate analysis of factors predicting OS.

Variable	Univariate analysis				Multivariate analysis			
	HR	95% CI		*P*	HR	95% CI		*P*
		Lower	Upper			Lower	Upper	
Age, years								
≥ 60 vs < 60	0.818	0.445	1.505	0.519				
Gender								
Female vs male	0.563	0.299	1.059	0.075	0.536	0.276	1.039	0.065
Primary tumor site								
center colon vs right colon	0.704	0.273	1.813	0.467				
Rectum vs right colon	0.697	0.280	1.738	0.439				
Stage at initial diagnosis								
Postoperative recurrence vs initial diagnosis of stage IV	0.819	0.449	1.495	0.516				
Number of metastatic sites								
Single vs multiple (≥ 2)	0.469	0.217	1.012	0.054	1.121	0.408	3.079	0.824
Lymph node metastasis								
No metastasis vs metastasis	0.435	0.235	0.807	0.008*	0.563	0.262	1.207	0.140
Liver metastasis								
No metastasis vs metastasis	0.410	0.221	0.763	0.005*	0.386	0.179	0.832	0.015*
Lung metastasis								
Metastasis vs no metastasis	0.595	0.327	1.084	0.090	0.722	0.325	1.602	0.423
Bone metastasis								
No metastasis vs metastasis	0.965	0.345	2.701	0.946				
Peritoneum metastasis								
No metastasis vs metastasis	0.559	0.295	1.059	0.074	0.555	0.251	1.230	0.147
RAS status								
Mutant vs wild	1.074	0.568	2.031	0.826				
Unknown vs wild	0.658	0.261	1.655	0.373				
BRAF status								
Mutant vs wild	0.612	0.146	2.569	0.502				
Unknown vs wild	0.839	0.435	1.619	0.601				

CI, confidence interval; HR, hazard ratio; OS, overall survival; TT group, targeted therapy group; TI group, targeted-immunotherapy combination group. * indicates statistical significance.

**Figure 2:**
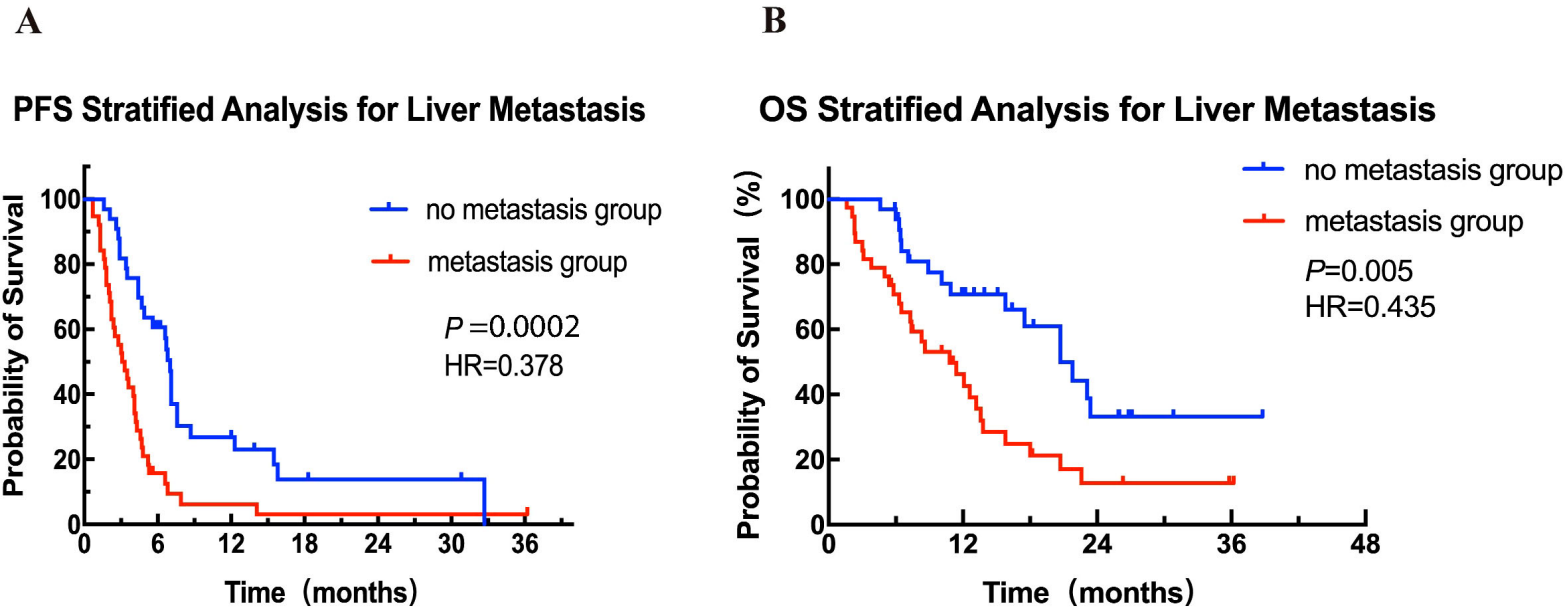
Kaplan-Meier curves for PFS **(A)** and OS **(B)** for patients with and without liver metastasis. HR, hazard ratio; OS, overall survival; PFS, progression-free survival.

Based on the risk factor liver metastasis, patients were stratified into liver metastasis group (n = 38) and non-liver metastasis group (n = 33) for further analysis. As shown in **Figure 2A**, patients in the non-liver metastasis group had a median PFS of 7 months (95% CI: 6.6-7.4), significantly better than the 3.2 months (95% CI: 2.0-4.2) observed in the liver metastasis group, with a statistically significant difference between the groups (HR = 0.39, 95% CI: 0.23-0.65, *P* = 0.0002). Similarly, a significant improved OS was observed in the non-liver metastasis group as compared to that in the liver metastasis group (median 20.7 months vs 10.8 months, HR = 0.41, 95% CI: 0.22-0.76, *P* = 0.005; **Figure 2B**).

**Figure 3:**
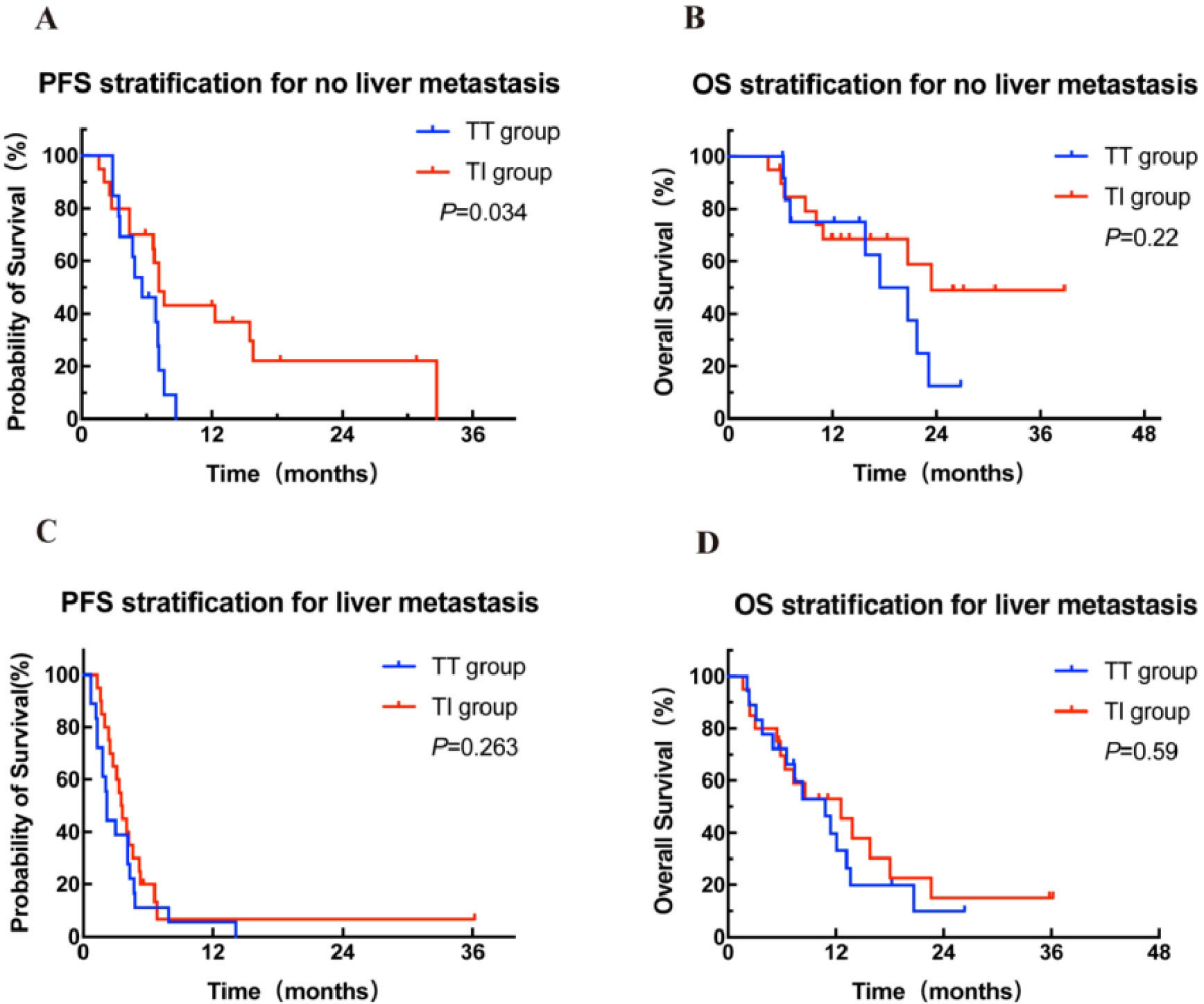
Kaplan-Meier curves for PFS **(A, C)** stratified by treatment modalities for patients with and without liver metastasis. Kaplan-Meier curves for OS **(B, D)** stratified by treatment modalities for patients with and without liver metastasis. OS, overall survival; PFS, progression-free survival; TT group, targeted therapy group; TI group, targeted-immunotherapy combination group.

Further stratified analysis of PFS and OS was performed in patients with and without liver metastases according to different treatment modalities. Among the 38 patients without liver metastases, the median PFS in the TI group (n = 20) was significantly superior to that in the TT group (n = 13) (7.1 months vs 5.6 months, HR = 0.42, 95% CI: 0.18-0.97, *P* = 0.034; **Figure 3A**), and an improvement trend in OS was observed in the TI group compared to the TT group (23.4 months vs 17.5 months, *P* = 0.22; **Figure 3B**). In contrast, in patients with liver metastases, there was no significant difference in either PFS or OS between the two treatment groups (**Figures 3C, D**).

**Figure 4:**
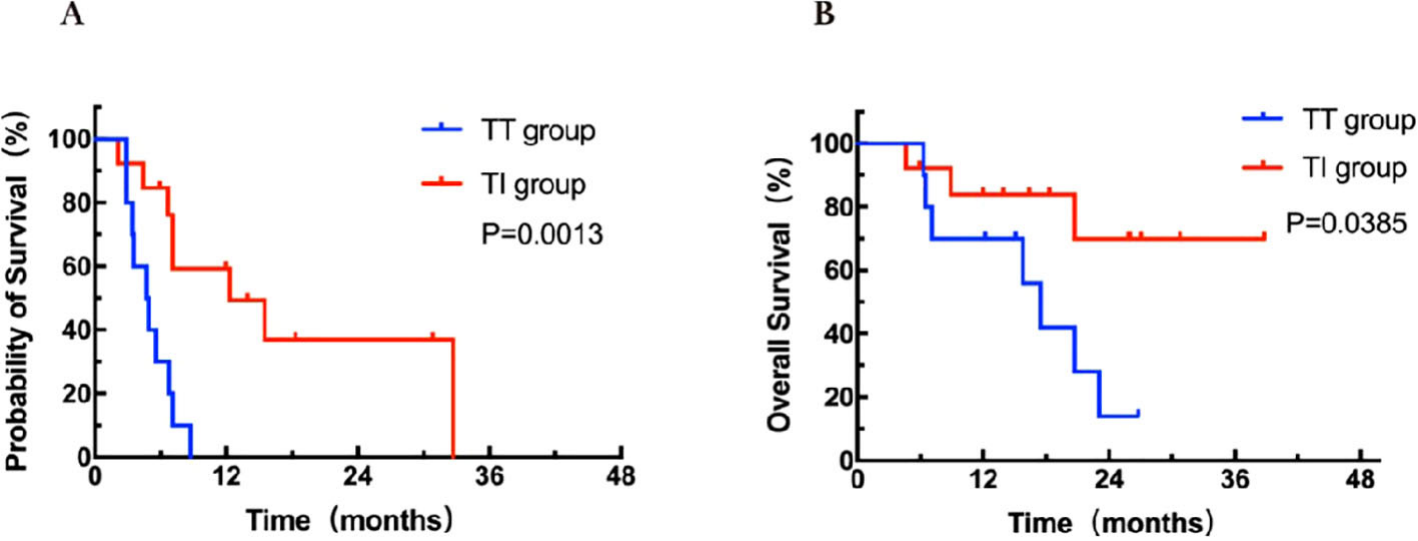
Kaplan-Meier curves for PFS **(A)** and OS **(B)** stratified by treatment modalities for patients with lung metastasis. OS, overall survival; PFS, progression-free survival; TT group, targeted therapy group; TI group, targeted-immunotherapy combination group.

In the 23 patients with only lung metastasis, there was a significant difference in PFS between the TT group (n = 10) and the TI group (n = 13) (4.7 months vs 12.3months, HR = 0.20, 95% CI: 9.8-25.3, *P* = 0.0013; **Figure 4A**). Patients in the TT group had a worse OS of 16.5 months compared to 31.1 months in the TI group (HR = 0.27, 95% CI: 11.8-21.2, *P* = 0.038; **Figure 4B**). Patients with only lung metastases may derive the greatest benefit from targeted-immunotherapy combination.

## 4 Discussion

Stratified therapy based on genetic testing is currently the main strategy for third-line treatment of mCRC. According to several large clinical trials, anti-PD-1 antibodies have been approved by the US Food and Drug Administration for the treatment of patients with MSI-H or dMMR mCRC (9, 18). However, for the vast majority of patients with MSS tumors, single-agent chemotherapy and immunotherapy are almost ineffective (8, 19). Currently, there are few trials on the efficacy and safety of targeted therapy combined with immune checkpoint inhibitors (ICIs) for MSS mCRC. Fruquintinib and regorafenib, both anti-angiogenic drugs, are third-line treatment options for mCRC (20, 21). Preclinical studies have shown synergistic effects of the combination of fruquintinib or regorafenib with PD-1 inhibitors in CRC models (22, 23). Meanwhile, some researchers believe that anti-angiogenic treatment may improve the immune condition of the tumor microenvironment, alleviate the immunosuppressive state, and thereby benefit immunotherapy (24). In this study, we conducted a retrospective analysis of the efficacy of targeted therapy alone versus targeted-immunotherapy combination in patients with MSS mCRC, identifying the potential beneficiary population for the targeted-immunotherapy combination.

In this study, the ORR was 12.7% and the DCR was 71.8% in the overall population. Among patients who received only targeted therapy, the ORR was 3.2% and the DCR was 58.1%; however, in those who received targeted therapy combined with immunotherapy, the ORR improved to 20% and the DCR to 82.5%. This indicates that the addition of immunotherapy enhances tumor response to regorafenib or fruquintinib in patients with MSS mCRC. The phase Ib REGONIVO trial (NCT03406871), which enrolled 24 patients with MSS mCRC, reported an ORR of 33% and a DCR of 88%, significantly surpassing our findings (16). This discrepancy might be attributed to the different types of ICIs used. The REGONIVO trial specifically explored the combination of nivolumab and regorafenib, whereas in real-world clinical practice, patients may receive a variety of ICIs. Our study included additional ICIs beyond nivolumab, such as the homemade agent sintilimab.

In our study, median PFS was 4.6 months and median OS was 15.8 months for all patients receiving targeted-immunotherapy combination. Standard third-line treatment regimens included chemotherapy or targeted therapies such as irinotecan combined with cetuximab, regorafenib, fruquintinib, and trifluridine/tipiracil (TAS-102) (25). Patients with refractory mCRC who received anti-angiogenic treatment had a median PFS of approximately 2 months and a median OS of 7 months (26, 27). Our results suggest that the combination strategy of targeted therapy and immunotherapy may have certain advantages over traditional therapies. Previous small-scale studies have also evaluated the efficacy of combining ICIs with regorafenib in MSS CRC (28). Based on these, for refractory MSS CRC, a combined strategy of targeted therapy and immunotherapy may represent an effective treatment option.

Not all patients with MSS CRC responded well to combined therapies, suggesting the need for further stratification of patient populations to improve survival benefits. To assist in patient selection, we conducted Cox regression analyses for the discernment of prognostic-related risk factors, further identifying clinical characteristics associated with the effectiveness of targeted-immunotherapy combination. Multivariable analysis revealed significant correlations between liver metastasis and both PFS and OS. Clinical data indicated that patients with liver metastases responded less favorably to anti-PD-1 antibodies than those without liver metastases, a finding supported by basic research (29). Our results aligned with prior studies that the presence of liver metastases was an independent poor prognostic factor for various cancers, particularly in the context of ICI therapy (30, 31). The liver metastatic microenvironment is typically considered to be immunosuppressive, characterized by diminished infiltration of CD8+ T cells and enriched functionality of immune escape pathways (32, 33). Furthermore, recent studies have suggested that liver metastases could induce systemic resistance to ICIs mediated by macrophages and regulatory T cells (29). In the REGOTORI study, patients with liver metastases had a lower ORR compared to those without liver metastases (8.7% vs 30.0%). Indeed, various studies have shown that liver metastasis could reduce the effectiveness of anti-PD-1 antibodies. In patients with melanoma or non-small cell lung cancer treated with pembrolizumab, the response rates were 56.3% in those without liver metastasis and 30.6% in those with liver metastasis. Additionally, liver metastasis was also associated with significantly shorter PFS, with a median of 5.1 months vs 20.1 months (31). Our current study showed that patients without liver metastases responded better and derived greater benefit from the combination of targeted therapy and immunotherapy. In our previous study, we conducted a comprehensive analysis of MSS CRC cases with extrahepatic metastases. The results showed that, although MSS CRC is still referred to as a “cold tumor” in this field, patients with non-liver metastatic MSS mCRC could still benefit from targeted-immunotherapy combination (34). Therefore, effective management of liver metastases may be a key to overcoming resistance to ICIs.

This study found that in patients with only lung metastases, there were significant differences in both PFS and OS between targeted therapy alone and targeted-immunotherapy combination (HR = 0.20 for PFS and HR = 0.27 for OS). This suggests that patients with only lung metastases may benefit most from targeted-immunotherapy combination. Meanwhile, significant differences in PFS and OS were observed in patients with various distant metastasis conditions and treatment modalities. In the FRESCO trial, regorafenib was reported to yield a radiological CR in one case of multiple lung metastases from ascending colon cancer. Of note, regorafenib is primarily approved for third-line therapy of mCRC patients, and detailed reports on its effectiveness in lung metastases are limited. The case discussed demonstrated that in some instances, regorafenib could lead to significant tumor reduction, suggesting its potential efficacy in mCRC with lung metastases (21, 35). The results of this particular case from the FRESCO trial were consistent with the findings of this study. This evidence highlights the need for personalized treatment strategies in mCRC, particularly considering the organ-specific impacts of therapies.

This study has several limitations. Firstly, the study adopted a retrospective design, which restricted the applicability of the findings. Secondly, there was no restriction on the therapeutic drugs used in the study, affecting the consistency of the treatment regimen. Thirdly, the number of patients included was small. Fourth, not all patients underwent RAS and BRAF genetic testing, limiting the analysis of their impact on the efficacy of the drugs. To overcome the limitations of the retrospective design, we are planning to conduct a larger study to improve statistical power, and will ensure that all patients undergo RAS and BRAF gene testing in order to comprehensively analyze the impact of genotype on drug efficacy.

## 5 Conclusion

Targeted-immunotherapy combination showed more benefit than targeted therapy alone in the third-line or beyond setting for MSS mCRC. Liver metastasis might be a key factor in the poor prognosis of this population. Patients with only lung metastasis were most likely to benefit from targeted-immunotherapy combination.

## Data Availability

The raw data supporting the conclusions of this article will be made available by the authors, without undue reservation.
